# Disentangling socioeconomic inequalities of type 2 diabetes mellitus in Chile: A population-based analysis

**DOI:** 10.1371/journal.pone.0238534

**Published:** 2020-09-03

**Authors:** Manuel S. Ortiz, Baltica Cabieses, Marcela Oyarte, Paula Repetto

**Affiliations:** 1 Departamento de Psicología, Universidad de La Frontera, Temuco, Chile; 2 Instituto de Ciencias e Innovación en Medicina (ICIM), Facultad de Medicina Clínica Alemana, Universidad del Desarrollo, Santiago, Chile; 3 Instituto de Salud Pública de Chile, Santiago, Chile; 4 Escuela de Psicología, Pontificia Universidad Católica de Chile, Santiago, Chile; Sciensano, BELGIUM

## Abstract

**Introduction:**

Chile experiences a growing prevalence of DM2 in its adult population over time. The country has prioritised the diagnosis and treatment of DM2 through a universal health care package, largely focused on the clinical dimensions of the disease. We analysed the significance of socioeconomic variables in the prevalence of DM2, as well as its related dimensions of presence of complications (diabetic foot and ophthalmologic complications), attendance to health checks and acquisition of recommended lifestyle changes due to this condition.

**Methods:**

Secondary analysis of the national health survey (ENS) 2016–2017 (n = 6,233 respondents). Crude and income-adjusted odds of reporting DM2 was estimated, as well as the relationship between complications due to diabetes and a number of clinical and sociodemographic variables using weighted log-linear multiple regression models.

**Results:**

We found a clear social gradient of the prevalence of DM2 by household income quintiles and educational level in the adult population. Income quintile and educational level gradients remained significantly associated with the presence of complications and attendance to health checks. We found no significant association, however; between income quintile and reported lifestyle change. The association between complications due to DM2 and socioeconomic variables, particularly income, remained relevant even after adjusting for all sociodemographic variables.

**Conclusion:**

This is the first study to analyse the association between DM2 and socioeconomic variables in Chile, useful for monitoring and policy planning. Income was strongly associated with DM2 prevalence and with related clinical variables (complications and attendance to health checks). Age, health care provision and educational level were also relevant factors, but lost significance in the fully adjusted model.

## Introduction

Type 2 Diabetes Mellitus (DM2) is a multi-systemic metabolic disorder, caused by a malfunction in the uptake or the secretion of insulin, leading to chronic hyperglycemia that over time puts the person at risk of specific macro and microvascular complications associated with the disease [1]. DM2 is an independent risk factor for cardiovascular diseases (CVD), doubling the risk of suffering from macro-vascular complications, coronary heart disease, stroke and peripheral arterial disease, which are responsible for most of the deaths in these patients [2]. It is also the main cause of diabetic retinopathy and disability. According to the latest report of the International Diabetes Federation, its incidence and prevalence continue to increase massively worldwide. Currently 387 million people have this disease and it is expected that by 2035 there will be a 55% increase in global prevalence, reaching 592 million people [3]. DM2 is one of the pathologies with the highest rates of premature death in most of the developed and developing countries, causing 5.1 million deaths in 2013 [3].

Chile is a high-income country with a Gross Domestic Product (GDP) per capita of $27 059 (USD) in 2019 [4]. It has a population of just over 18 million inhabitants and in recent decades has experienced major economic and demographic changes, moving towards an emerging high-income country [5,6]. Hence, there is evidence from this country suggesting good general health status of the population, but with a growing burden of chronic conditions in an aging nation [5,7]. Positive global health indicators in Chile could be the consequence of major public health initiatives conducted in the past century, firstly focused primarily on maternal-infant mortality and infectious epidemics and more recently on chronic diseases and cancer [8,9].

Despite this significant accomplishments, socioeconomic inequality remains a large problem. That is, not all socioeconomic groups in the country have profited from its growth and development in the same way [6]. Inequalities in the health status of Chileans varies significantly by social and demographic indicators like type of healthcare system [10], gender [11], age groups [9], and the structure of the health care system [12,13]. These systematic and unfair differences in social status, living conditions and health outcomes have been the main argument for the recent profound social crisis in the country faced in past October 2019. As stated by several experts, despite the country´s intense economic growth in the past three decades, the unfair distribution of wealth and prosperity has affected not only the social image and international prestige of the country, but also its capacity to improve the unequal distribution of most health outcomes in the population, systematically affecting those in greater socioeconomic adversity.

The unequal distribution of health outcomes by socioeconomic status in Chile is also true for DM2. When comparing the National Health Survey (ENS) of 2003 with that of 2009–2010, there is an increase in prevalence from 6.3% to 9.4%, respectively [6]. Unlike what is described internationally, where half of those with the disease do not know their condition, in Chile according to data from the ENS 2009–2010 85% of the people who have DM know it. However, only 34.32% of those affected achieve HbA1c figures less than 7%. The ENS 2003 also showed a significant socioeconomic gradient of the prevalence of DM2 in the adult population. That is, there was a 21.3% prevalence of DM2 in the population without any education, compared to 2.5% prevalence among those with higher education [14]. Differences in presence of complications, attendance to health checks and acquisition of recommended lifestyle changes by socioeconomic status in DM2 patients has not been analysed using the national health surveys. This information would be useful for chronic disease monitoring and assessment of health care innovations that aim at reducing the gaps in adequate management of DM2 in the adult population in Chile.

In response to sustainable development goal 10 related to reduce inequality within and among countries, and to the global burden of DM2, novel research on the complex relationship between DM2 and socioeconomic inequality is urgently required. Chile experiences a growing prevalence of DM2 in its adult population over time. For this reason, the country has prioritized the diagnosis and treatment of DM2 through a universal package that is part of the Explicit Health Guarantees list of the country. These relevant improvements are largely focused on the clinical dimensions of the disease; however, a deeper understanding of its social determinants is less interrogated and considered. Therefore, Chile needs to improve its comprehension of how social variables interact with the prevalence of DM2, as well as its related dimensions of presence of complications, periodicity of health checks and acquisition of recommended lifestyle changes secondary to this condition. Reducing socioeconomic inequalities in health, including DM2, could have a vast effect in measures of quality of life, premature death and broader relevant societal measures like the sense of social injustice in population health. Therefore, the purpose of this study was to (i) estimate the prevalence of DM2 in adults in Chile by adjusting for a series of sociodemographic variables, and (ii) analyse the relationship between these variables and the presence of complications, attendance to health checks and acquisition of recommended lifestyle changes due to DM2.

## Methods

Cross-sectional study, based on the secondary analysis of the national health survey (ENS) 2016–2017, freely available in its anonymised version. From this, the prevalence of diabetes was estimated by adjusting for a series of sociodemographic variables, and the relationship between these variables and the presence of complications (diabetic foot and ophthalmologic complications) due to diabetes, periodic health checks and lifestyle changes due to diabetes was also analysed.

### Data source

The National Health Survey (ENS) focuses on the estimation of diseases and treatments that are receiving men and women of 15 years or older, Chilean or foreigners, who reside in rural and urban settings in Chile. Since 2009 this survey has covered more than 40 health conditions or prioritised diseases, along with an extensive list of risk factors, protectors and socioeconomic determinants. This national survey has been conducted since 2003 and is a valuable support for the formulation of prevention and public health plans for the country.

The ENS 2016–2017 corresponds to a cross-sectional population survey, which used a complex probabilistic sampling, geographically stratified, allowing representativeness at national, regional and geographic (urban / rural) levels. The survey considers a main sample, the “ENS” sample, and three secondary samples: the mental health subsample, the laboratory exam subsample (blood and urine), and the iodine subsample. Data collection was carried out between August 2016 and March 2017 based on a questionnaire applied by a trained interviewer and biometric measurements carried out by a trained health professional.

The sample size of the ENS 2016–2017 was 6,233 respondents. Of these, 5,520 had laboratory tests according to protocol and 886 said that ever in their life a doctor, a nurse or another health professional told him that he/she has had, has or suffers from DM2 (72 said that it happened during pregnancy). The survey had an absolute sampling error of 2.6% nationwide, a household response rate of 66% (67% of eligible) and a total participation rate of 90.2%.

### Variables

DM2 prevalence: We included all those who answered “yes” to the question “Have you ever been told by a doctor, nurse or other health professional to tell you that you have had or have or suffer from diabetes (high blood sugar)?” Participants who claimed that this occurred during pregnancy were discarded.

Complications secondary to DM2: We created this variable from the following two questions: (i) “In the last year, have you had to consult a health professional or attend a cure for “ulcers, wounds or sores” that do not close, or that do not heal on the legs or feet (or "gangrene")?”; and (ii) "Have you ever been told by a doctor that you have an alteration of the retina of the eye or that you have retinopathy due to diabetes?" The complications secondary to DM2 indicator was constructed, considering as complications those who answered “yes” to one or both questions. The response categories of these variable were: (i) yes, diabetic foot, (ii) yes, diabetic retinopathy, (iii) yes, both diabetic foot and diabetic; and (iv) no, without complications.

Attendance to health checks in past year: This indicator was constructed from the following questions: (i) “Because of your diabetes or high blood sugar, did you attend a physician for a health check in the last year?”; (ii) "Because of his diabetes or high blood sugar, did you attend a nurse for a health check in the last year?"; and (iii) "Because of his diabetes or high blood sugar, did you attend a nutritionist for a health check in the last year?". The variable had the following response categories: (i) yes, medical only control, (ii) yes, only nurse, (iii) yes, only nutritionist, (iv) yes, doctor and nurse, (v) yes, doctor and nutritionist, (vi) yes, nutritionist and nurse, (vii) yes, doctor, nurse and nutritionist, (viii) no, without any health check in the last year.

Acquisition of recommended lifestyle changes: The change in lifestyle corresponded to the question “Have you ever done any program, treatment change in lifestyle (diet, exercise or weight loss) for diabetes or high blood sugar?" of the poll. Response categories: yes/no.

Socioeconomic and demographic variables: sex, age (continuous and ranges: 15–24, 25–44, 45–64, >64), area of residence (urban/rural), aboriginal ethnic belonging (to any of the 9 aboriginal groups recognized by law in Chile), number of people per household, type of health care provision (Public: Fonasa, Private: Isapre, other, none/don´t know), educational level (no studies, basic, medium, higher), overcrowding of housing (with/without) and household income quintile.

### Data analysis

The descriptive statistics of DM2 prevalence, presence of complications (diabetic foot and ophthalmologic complications), periodic control and lifestyle changes, was performed for the total population and stratified by socioeconomic and demographic variables, using proportions with their respective confidence intervals (Chi-square tests included in order to look at differences in dependent variables by categories of independent variables of interest).

For the analysis of the relationship between socioeconomic/demographic variables and outcomes of interest, the variables complications and attendance to health checks were dichotomized as follows: yes, if any and no, if none. For crude association analysis we estimated logistic regression models and for adjusted analysis we estimated log-linear models. In order to attain all relevant variables in a single model, we modelled the odds of reporting complications secondary to DM2, in which attendance to health checks and changes in lifestyle were added as co-variables. First, models were adjusted considering income quintile only; then we included all other sociodemographic variables. In order to obtain a better fit of the data, we estimated a number of log-linear regressions for complications secondary to DM2 as the main outcome variable, as follows: Model 1 adjusted by attendance to health checks only, Model 2 adjusted by change in lifestyle only, Model 3 adjusted by both, Model 4 added income quintiles, and Model 5 included all study variables.

Analyses were performed considering the complex nature of the sample, applying the weighting factor variable, clusters and strata and using Taylor's linearization method, with 95% confidence and a significance of 0.05. Analyses were conducted in Stata 16.

## Results

A total of 814 respondents, representing a weighted sample size of 1,468,183 people, said that at some time a doctor, nurse or other health professional told him or her that suffers from DM2, excluding cases detected during pregnancy, corresponding to 10.1% (95% CI: 8.9% - 11.4%). Among this group, 10.8% (95% CI: 8.1% - 14.2%) said they had to consult a health professional for leg or foot injuries or altered eye retina. On the other hand, 80.7% (95% CI: 75.1% - 85.2%) of the DM2 patients ever did some life change program, while 83.9% (95% CI: 78.9% - 87.8%) attended a health check in the past year (doctor, nurse or nutritionist) (Table 1).

**Table 1:** Prevalence of DM2 and related clinical variables in adult population in Chile, ENS 2016–2017. Weighted statistics.

	N	%*	95% Confidence Intervals
DM2 yes	1,468,183	10.11%	(8.92% - 11,44%)
Complications secondary to DM2	159,378	10.86%	(8.19% - 14.25%)
Yes, diabetic foot	80,212	5.46%	(3.69% - 8.03%)
Yes, diabetic retinopathy	59,212	4.03%	(2.60% - 6.21%)
Yes, both	19,954	1.36%	(0.49% - 3.71%)
Attendance to heath checks in past year	1,231,775	83.90%	(78.95% - 87.86%)
Yes, doctor	258,196	17.59%	(12.99% - 23.37%)
Yes, nurse	11,636	0.79%	(0.22% - 2.87%)
Yes, nutricionist	1,838	0.13%	(0.02% - 0.75%)
Yes, doctor and nurse	155,768	10.61%	(7.47% - 14.85%)
Yes, doctor and nutritionist	48,119	3.28%	(2.00% - 5.34%)
Yes, nurse and nutricionist	2,536	0.17%	(0.07% - 0.44%)
Yes, doctor nurse and nutritionist	753,682	51.33%	(44.56% -58.06%)
Change in lifestyle yes	1,184,999	80,71%	(75.14% - 85.28%)

**Fig 1:**
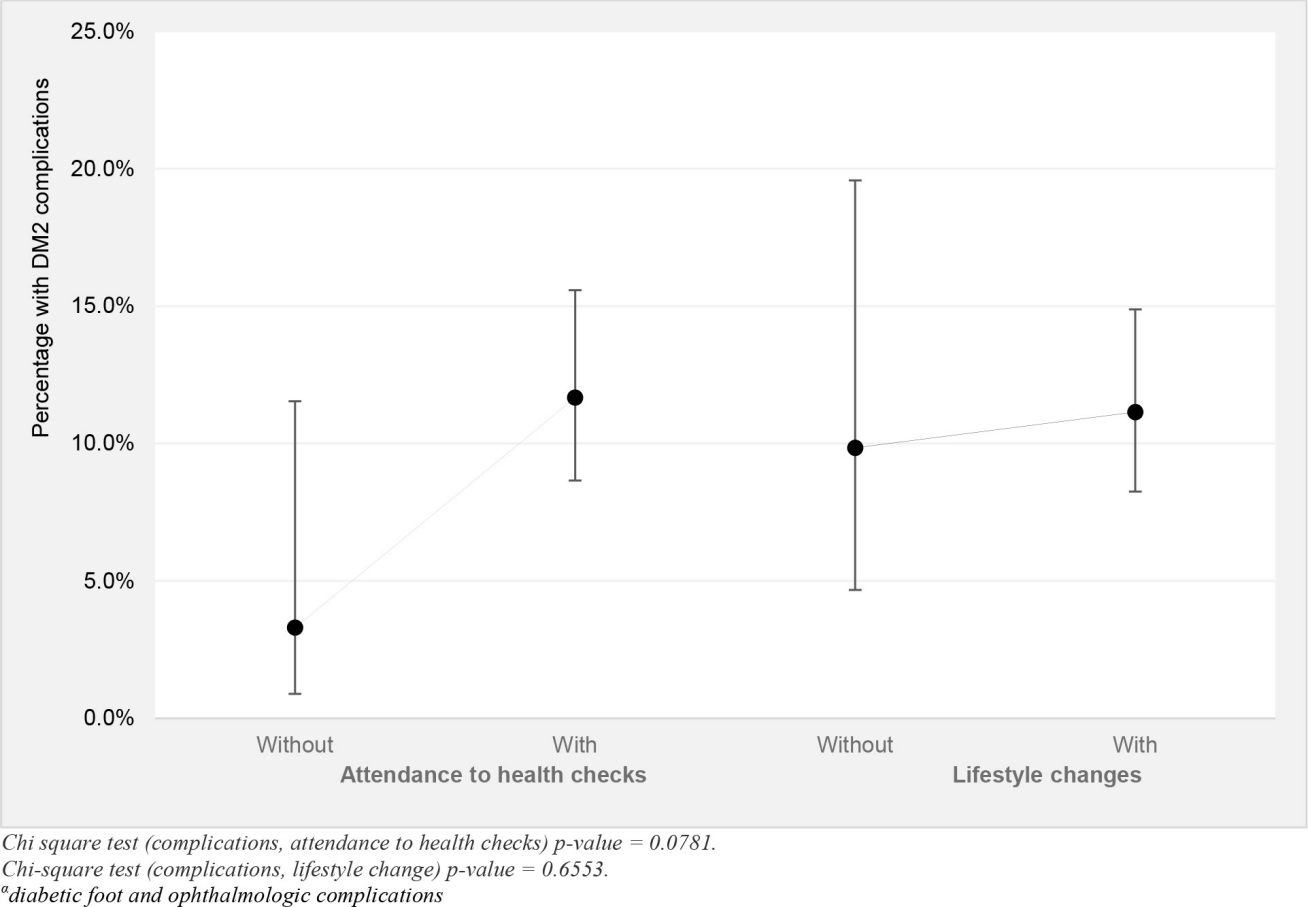
Differences in reported complications^α^ secondary to DM2 in adult population who did and did not attend health checks in past year and changed their lifestyle. ENS 2016–2017. Weighted statistics.

Participants with DM2 without periodic health checks (medical, nurse or nutritionist) reported a higher proportion of complications due to diabetes (diabetic foot and ophthalmologic complications) than those who attended any health check in the past year (11.1% vs. 9.8%). The same was observed for those who did not change their lifestyle in contrast to those who did change their lifestyle (3.3% vs. 11.6%). In both cases, however, the differences were not statistically significant (Fig 1).

When analysing socioeconomic variables such as income quintile, educational level and overcrowding, a social gradient is observed in the prevalence of DM2. The lower-income quintiles had a higher prevalence of DM2 than the top higher-income quintiles (13.8% Quintile I vs. 8.5% Quintile V). This social gradient per income quintile was not significant in most categories, since the confidence intervals of quintile I and IV only did not overlap. Similarly, as the educational level increased, the proportion of the population with DM2 decreased (30% in people who never attended an educational institution vs 6.1% in people with higher education). In terms of complications due to the disease, the poorest quintile and people who have never attended school presented complications in a higher percentage than the richest quintile and people with higher education, respectively. A significant association (p-value <0.05) was observed between the prevalence of DM2/complications associated with DM2 and the socioeconomic variables income quintile and educational level. In the case of attendance to health checks in past year and life changes, there was no significant association with socioeconomic variables. In all cases, age was a significant factor, where at a higher age there was a higher prevalence of DM2, a higher percentage of complications, and a higher proportion of attendance to health checks (Table 2).

**Table 2:** Prevalence of DM2 and related clinical variables by sociodemographic characteristics in adult population in Chile, ENS 2016–2017. Weighted statistics.

		DM2		Complications		Attendance to health checks		Change in lifestyle	
		%	IC95%	%	IC95%	%	IC95%	%	IC95%
Income quintile	I (poorest)	13.8%	(11.3% - 16.9%)	16,6%	(10,5% - 25.4%)	94.7%	(90.5% - 97.1%)	73.9%	(60.6% - 83.9%)
	II	10.0%	(7.7% - 12.9%)	7,4%	(4,2% - 12.7%)	81.7%	(69.5% - 89.7%)	83.2%	(74.2% - 89.5%)
	III	9.7%	(7.0% - 13.4%)	12,4%	(4,8% - 28.4%)	86.5%	(74.3% - 93.4%)	82.3%	(69.9% - 90.3%)
	IV	6.9%	(4.9% - 9.5%)	12,3%	(5,7% - 24.8%)	82.1%	(64.7% - 92.0%)	92.6%	(84.0% - 96.7%)
	V (wealthiest)	8.5%	(5.7% - 12.5%)	0,9%	(0,2% - 3.4%)	78.2%	(60.1% - 89.6%)	80.3%	(59.0% - 92.0%)
Educational level	Never attended	30.0%	(16.41% - 48.3%)	14,6%	(4,6% - 37.5%)	91.3%	(61.1% - 98.6%)	37.5%	(14.8% - 67.5%)
	Primary	16.4%	(14.0% - 19.2%)	14,8%	(10,2% - 20.8%)	88.3%	(80.9% - 93.1%)	82.8%	(76.3% - 87.7%)
	Secondary	8.8%	(7.2% - 10.8%)	10,8%	(6,4% - 17.8%)	82.1%	(73.4% - 88.5%)	78.0%	(69.4% - 84.7%)
	Higher	6.1%	(4.4% - 8.3%)	2,1%	(0,7% - 6.5%)	77.3%	(62.9% - 87.2%)	92.8%	(82.3% - 97.3%)
	Do not know/not respond	19.9%	(7.2% - 44.4%)	0,0%	-	79.7%	(34.6% - 96.7%)	97.5%	(77.6% - 99.8%)
Household	Yes	10.5%	(9.3% - 11.9%)	11,2%	(8,4% - 14.8%)	84.7%	(80.0% - 88.5%)	81.6%	(76.8% - 85.7%)
overcrowding	No	6.4%	(2.9% - 13.2%)	3,9%	(0,7% - 20.4%)	67.8%	(28.3% - 91.8%)	62.3%	(22.6% - 90.3%)
Health care provision	Public: Fonasa	10.0%	(8.8% - 11.4%)	13,2%	(10,1% - 17.2%)	83.7%	(78.3% - 88.0%)	80.5%	(74.2% - 85.6%)
	Private: Isapre	13.0%	(8.6% - 19.0%)	1,7%	(0,4% - 6.3%)	88.4%	(73.6% - 95.4%)	80.8%	(59.7% - 92.2%)
	Other	11.8%	(5.4% - 24.0%)	5,2%	(0,7% - 29.3%)	98.3%	(90.9% - 99.7%)	95.3%	(83.2% - 98.8%)
	None	5.0%	(2.6% - 9.5%)	5,7%	(1,2% - 23.9%)	54.4%	(24.0% - 81.9%)	75.4%	(37.6% - 93.9%)
Number of household	1	14.3%	(11.9% - 17.2%)	14,6%	(9,8% - 21.1%)	79.8%	(68.1% - 88.0%)	71.4%	(59.8% - 80.8%)
members	2	14.2%	(11.4% - 17.7%)	9,3%	(4,9% - 17.1%)	88.7%	(81.2% - 93.5%)	85.7%	(74.2% - 92.6%)
	3 or more	8.5%	(7.1% - 10.3%)	10,6%	(7,2% - 15.5%)	82.9%	(75.5% - 88.4%)	80.8%	(72.8% - 86.9%)
Sex	Men	8.5%	(6.9% - 10.3%)	11,0%	(6,9% - 17.1%)	79.4%	(70.5% - 86.2%)	82.2%	(72.2% - 89.1%)
	Women	11.7%	(10.1% - 13.5%)	10,8%	(7,5% - 15.2%)	87.1%	(81.3% - 91.2%)	79.7%	(71.8% - 85.8%)
Age range	15–24	1.4%	(0.7% - 2.7%)	0,0%	-	56.7%	(30.4% - 79.7%)	85.8%	(61.2% - 95.9%)
	25–44	5.3%	(3.7% - 7.5%)	2,5%	(0,8% - 7.3%)	68.1%	(50.5% - 81.6%)	64.4%	(46.6% - 78.9%)
	45–64	14.1%	(11.8% - 16.9%)	7,4%	(4,4% - 12.2%)	89.6%	(84.1% - 93.4%)	87.4%	(81.5% - 91.6%)
	65 or more	26.7%	(22.9% - 31.0%)	20,5%	(14,8% - 27.7%)	87.8%	(81.2% - 92.3%)	81.3%	(72.9% - 87.6%)
Zone	Urban	10.2%	(8.9% - 11.7%)	10,5%	(7,7% - 14.2%)	83.8%	(78.4% - 88.1%)	80.4%	(74.3% - 85.4%)
	Rural	9.3%	(7.1% - 12.1%)	13,7%	(7,7% - 23.1%)	84.5%	(74.6% - 91.0%)	83.1%	(73.3% - 89.8%)
Aboriginal	No	10.2%	(9.0% - 11.6%)	11,2%	(8,3% - 14.9%)	83.6%	(78.3% - 87.8%)	81.8%	(76.8% - 86.0%)
ethnic group	Yes	9.1%	(5.8% - 13.8%)	6,9%	(2,5% - 17.6%)	87.9%	(74.0% - 94.9%)	67.2%	(38.6% - 87.0%)

*Percentages were obtained considering the “*Do not know/not respond*” category*. *P-value*: *Chi-square test (Diabetes)*: *Quintile 0*.*0001*, *Educational level 0*.*0000*, *Overcrowding 0*.*3885*, *health care provision 0*.*3719*, *number of household members 0*.*0001*, *sex 0*.*0022*, *age 0*.*0000*, *zone 0*.*7562*, *ethnic group 0*.*8736*. *Chi square test (Complications)*: *Quintile 0*.*0494*, *Educational level 0*.*0000*, *Overcrowding 0*.*4386*, *health care provision 0*.*0548*, *number of household members 0*.*0378*, *sex 0*.*8088*, *age 0*.*0000*, *zone 0*.*2225*, *ethnic group 0*.*4664*. *Chi square test (Periodic checks)*: *Quintile 0*.*1537*, *Educational level 0*.*5212*, *overcrowding 0*.*3500*, *health care provision 0*.*0214*, *number of household members 0*.*0083*, *sex 0*.*1888*, *age 0*.*0002*, *zone 0*, *1726*, *ethnic group 0*.*4294*. *Chi square test (Life change)*: *Quintile 0*.*5856*, *Educational level 0*.*0099*, *overcrowding 0*.*2920*, *health care provision 0*.*8559*, *number of household members 0*.*1184*, *sex 0*.*5764*, *age 0*.*0160*, *area 0*.*2822*, *ethnic group 0*.*2169*

In relation to healthcare provision, we found no significant difference in the prevalence of DM2, attendance to health checks in past year or change in lifestyle between public and private insurance. However, we did find a significantly lower proportion of people with DM2 who reported a complication secondary to the condition in the private healthcare compared to the public system (1.7% (95% CI: 0.4%– 6.3%) vs 13.2% (95% CI: 10.1%– 17.2%), respectively). There was no significant difference by number of household members, sex, urban versus rural area, and aboriginal ethnic belonging (Table 2).

**Fig 2:**
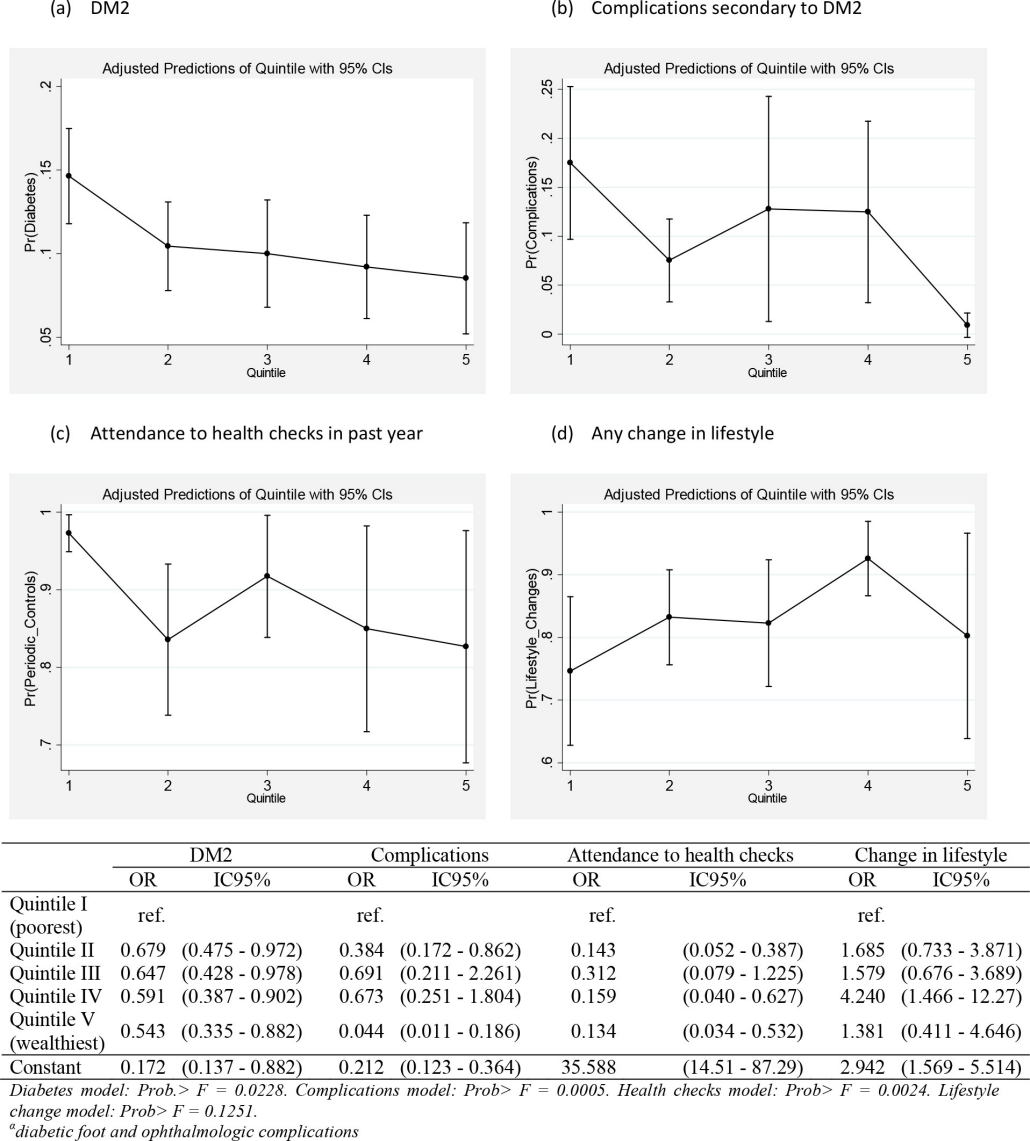
Crude Odds Ratio (OR) of reporting DM2, complications^α^ secondary to DM2, attendance to health checks and change in lifestyle by household income quintile. ENS 2016–2017. Weighted logistic regression models.

When analysing the association between each outcome variable (prevalence of DM2, complications, attendance to health checks and changes in lifestyle) and income quintile (crude logistic regression analysis), we observed a clear fine social gradient of the prevalence of DM2. Every income quintile had a lower odds of reporting DM2 compared to the bottom poorest income quintile (p<0.05). Income quintile gradients were less clear for the other outcomes but remained significantly associated with the presence of complications (diabetic foot and ophthalmologic complications) and attendance to health checks in many of the income quintile categories. We found no significant association, however; between income quintile and lifestyle change in the adult population under study (Fig 2).

Sequential multivariate regressions for complications secondary to DM2 (diabetic foot and ophthalmologic complications) as the main outcome variable were conducted (more detail in methods section). We found a consistent significant association between complications of DM2 and income quintile, with a social gradient favouring fewer complications among those in the top wealthiest quintile. This gradient was interpreted from the comparison of each category of income quintile to the reference wealthiest quintile, in which case all categories -except for the second poorest income- increased its magnitude of association to complications secondary to DM2. That is, the poorer the income quintile, the higher the magnitude of the odds of reporting complications due to DM2 in the adult population in Chile compared to the wealthiest income quintile, except for quintile 2 that did not follow this pattern. This gradient was maintained after adjusting for all sociodemographic variables, yet the magnitude of these associations decreased as well as its statistical significance. Age and a higher educational level remained significantly associated to complications due to DM2 in the fully adjusted model (Table 3).

**Table 3:** Adjusted Odds Ratio (OR) of reporting complicationsα secondary to DM2, by clinical variables and sociodemographic factors. ENS 2016–2017. Weighted log-linear regression models.

		Complications secondary to DM2						
		Model 1 OR (95%CI)	Model 2 OR (95%CI)	Model 3 OR (95%CI)	Model 4 OR (95%CI)	p-value	Model 5 OR (95%CI)	p-value
Attendance	Yes	3.619		3.637	4.440	0.070	3.798	0.114
to health checks		(IC:1.0–13.7)		(IC:1,0–13,8)	(IC:0.9–22.3)		(IC:0.7–19.9)	
	No	ref.		ref.	ref.		ref.	
Change in lifestyle	Yes		1.137	0.937	1.056	0.904	0.937	0.879
			(IC:0.5–2.5)	(IC:0.5–2.0)	(IC:0.4–2.6)		(IC:0.4–2.2)	
	No		ref.	ref.	ref.		ref.	
Income quintile	I (poorest)				16.065*	0.000	*8.755	0.030
					(IC:3.9–66.0)		(IC:1.2–62.1)	
	II				7.757*	0.007	3.934	0.172
					(IC:1,8–34.1)		(IC:0.6–28.1)	
	III				12.784*	0.001	*7.144	0.047
					(IC:2.7–60.8)		(IC:1.03–49.6)	
	IV				10.366*	0.004	6.091	0.054
					(IC:2.2–49.9)		(IC:1.0–38.4)	
	V (wealthiest)				ref.		ref.	
Educational level	Never attended						ref.	
	Primary						0.863 (IC:0.3–2.7)	0.802
	Secondary						1.029 (IC:0.4–4.7)	0.663
	Higher						0.757 (IC:0.1–4.2)	0.749
	NR						*0.000 (IC:0.0–0.0)	0.000
Overcrowding	Yes						0.696 (IC:0.1–8.8)	0.779
	No						ref.	
Health care	Public: Fonasa						2.813 (IC:0.3–25.3)	0.356
provision	Other						3.417 (IC:0.2–52.5)	0.377
	NR						1.873 (IC:0.1–36.1)	0.677
	Private: Isapre						ref.	
Number of	1						ref.	
household members	2						0.625 (IC:0.3–1.4)	0.260
	3 or more						1.044 (IC:0.5–2.0)	0.896
Sex	Men						ref.	
	Women						0.714 (IC:0.3–1.5)	0.363
Age (continuous)							*1.019 (IC:1.0–1.0)	0.040
Zone	Urban						ref.	
	Rural						1.127 (IC:0.6–2.2)	0.717
Aboriginal	No						ref.	
ethnic belonging	Yes						0.437 (IC:0.3–1.5)	0.398

*p-value<0.01 at 95% confidence.

^α^diabetic foot and ophthalmologic complications. NR: Do not know/not respond

## Discussion

The purpose of this study was to estimate the prevalence of DM2 in adults in Chile by adjusting for a series of sociodemographic variables, and to analyse the relationship between these variables and the presence of complications (diabetic foot and ophthalmologic complications), attendance to health checks and acquisition of recommended lifestyle changes due to DM2. In our study, we found a social gradient of the prevalence of DM2 by household income quintiles in the adult population. Income quintile gradients were less clear for the other outcomes but remained significantly associated with the presence of complications and attendance to health checks. We found no significant association between income quintile and reported lifestyle change; however, the lifestyle change question was very ample and could have not adequately represented a number of possible changes in lifestyle in this population. This significant association between complications due to DM2 and socioeconomic variables, particularly income, remained relevant even after adjusting for all sociodemographic variables included in this study. Ethnic belonging was not significantly associated with dependent variables of interest, which could respond to underrepresentation of this population in the survey.

In terms of DM2 prevalence, given its significant prevalence and high burden of disease, there is a global interest on this chronic condition [15]. International evidence demonstrates that crucial indicators like household income and head of the household´s educational level and occupational status have an inverse significant association with obesity and DM2 [e.g. 16,17]. Moreover, environmental indicators of socioeconomic deprivation and inequality are also pervasive in their association to DM2. As reported by previous research [17], neighbourhood socioeconomic disadvantage is associated with differences in health risks across the life course, including detrimental lifestyle factors from childhood onwards and worse glucose metabolism in adulthood. The unequal distribution of risk factors for DM2 by household and neighbour socioeconomic status yields structural health gaps within populations that are very challenging to modify and reverse, prevailing even across generations [18].

We found a significant association between socioeconomic variables (income, education) and complications (diabetic foot and ophthalmologic complications) and attendance to health checks. This adds to existing knowledge on the topic in Chile, as individual-level risk factors had been already identified, lacking analysis from a social determinants approach. Leiva et al. [19] recently reported that the main non-modifiable risk factors associated with diabetes in Chile were age ≥45 year, female and family history of diabetes; whereas the main modifiable risk factors were hypertension, overweight, obesity, central obesity, physical inactivity and higher levels of sitting time. A different study had looked at socioeconomic status but focused on the spatial clustering of Type 1 diabetes in children [20]. Similarly, an older study about socioeconomic factors associated with diabetes in adolescents in Chile reported that students coming from families in which the father had only primary school education, were significantly more likely to report having diabetes (odds ratio = 2.03 confidence intervals 1.02–4.04) [21]. More recently, Gonzalez-Agüero and colleagues [22] followed the health care trajectories of adolescents with diabetes in Chile and found that the implementation of a universal health coverage (UHC) plan for diabetes in the country did not lead to the promised equitable health care delivery for these patients. These findings are useful from a life course approach, as they inform about poor implementation of UHC for diabetes in Chile from childhood onwards. Public-private fragmentation of the Chilean healthcare system, relevant to Gonzalez-Agudelo´s study, also proved to be relevant in this study based on adult population with DM2 in Chile. We found that a lower proportion of people with DM2 in the private healthcare system reported a complication secondary to the condition compared to those who have access to the public healthcare system. Poorer health outcomes in the public system compared to the private system in Chile has been documented for several health outcomes in the past. This has been explained by differences in budget, specialists, technology, and demographics of beneficiaries, i.e. the private largely composed by the wealthiest and the youngest and the public by the oldest, poorest, and sickest(10.13). Hence, equitable health care delivery for all diabetic patients in Chile irrespective of type of healthcare provision, paediatric and adult, remains unaccomplished.

Study strengths are that we used a nationally-representative survey with acceptable response rate; we conducted standard statistical analysis for epidemiological analysis, which considered a number of socioeconomic variables widely used in the scientific literature and robust in the Chilean sociocultural context; we included four outcome variables related to DM2, prevalence and other three: complications (diabetic foot and ophthalmologic complications), attendance to heath checks and changes in lifestyle, which added complexity and unique information to the analysis. We recognise as limitations of this study its cross-sectional nature that does not allow for longitudinal analysis of variables of interest (i.e. we cannot say that income is a precursor of DM2, but we could test its association in our population); the non-weighted sample size was relatively small in some response categories, which produced large confidence intervals in some variables of interest; and there was a small sample representation of other social groups relevant in Chile like international migrants, ethnic groups and sexually diverse populations, restricting our chance to analyse these groups separately. Some study variables were very general and did not allow us to conduct a more detailed examination of their association with dependent variables (i.e. complications secondary to DM2, health attendance measured as annual check-up and lifestyle changes). We also lacked other variables like family and social support, and physical activity, which have proven their relevance in previous studies in the country [23,24]. Also, our analysis of the social gradient was largely descriptive using the wealthiest income quintile as reference. Future studies could expand this by using other analytical tools that better represent the social gradient, such as the concentration index and others.

Given its significant prevalence and high burden of disease, there is a global interest on DM2 [15]. This is a widespread public health problem that requires urgent action to develop effective prevention strategies [25]. Understanding environments in which populations develop DM2 over their lifetime is the key to such effective solutions, but these environments are complex and multidimensional. As recognised by the American Diabetes Association (ADA) new guidelines [26], DM2 is the result from a life-long trajectory of unhealthy environments, which include poor diet, sedentary lifestyle, cultural and social norms, food policies and food availability, and others. Undoubtedly, these influential environments tend to concentrate in areas of lower socioeconomic status in every country [27]. Socioeconomic inequalities are related to pro-DM2 environments at country and region levels and, therefore, could have a more explicit consideration in policy and practice from young ages [16,17,27]. Future research and policy analysis should take place about the complex relationship between socioeconomic inequality and the development of DM2 during childhood for a timely and effective prevention and management throughout the life course. Reducing socioeconomic inequalities within societies could not only contribute to the prevention and control of DM2, but also to many other non-communicable diseases and social issues that are strongly related to poor quality of life, premature death and social injustice.

Study findings are relevant to policy and practice. In terms of policy, the universal coverage plan for DM2 in the country, as part of the list of prioritised Explicit Health Guarantees, has represented a valuable effort from the health care system. According to our findings, it has proven acceptable attendance to health checks in the DM2 adult population in the past year (over 80% overall) and self-reported changes in lifestyle (80% reported a change). However, it has not been so effective in the unequal distribution of DM2 by income of educational level, as well as the prevalence of complications secondary to the condition. We need to understand that social dimensions of chronic conditions are influential in their appearance and evolution and, therefore, need targeted solutions for those sub-groups in higher risk and burden. The same in terms of clinical practices, novel solutions and interventions for DM2 populations must address differences in social and cultural norms, diet and physical activity, expectations and beliefs that are socially graded and stratified. Clinical practice for DM2 patients focused on individual-risk behaviours and biological dimensions of the disease will always be short-sighted in terms of broader social determinants of DM2 and chronic conditions. Future studies could continue to develop which social variables are relevant to each local context and how to address them in clinical practice for DM2 patients.
